# How do different lipid peroxidation mechanisms contribute to ferroptosis?

**DOI:** 10.1016/j.xcrp.2024.101932

**Published:** 2024-04-01

**Authors:** Quynh Do, Libin Xu

In the originally published version of this article, there were errors in the structures of compounds 2, 3, 4, 5, and 6 in Figure 3D. These structures have been redrawn correctly in the revised Figure 3, which can be seen below along with the original figure.

The authors regret this error.

## Figures and Tables

**Figure 3.**
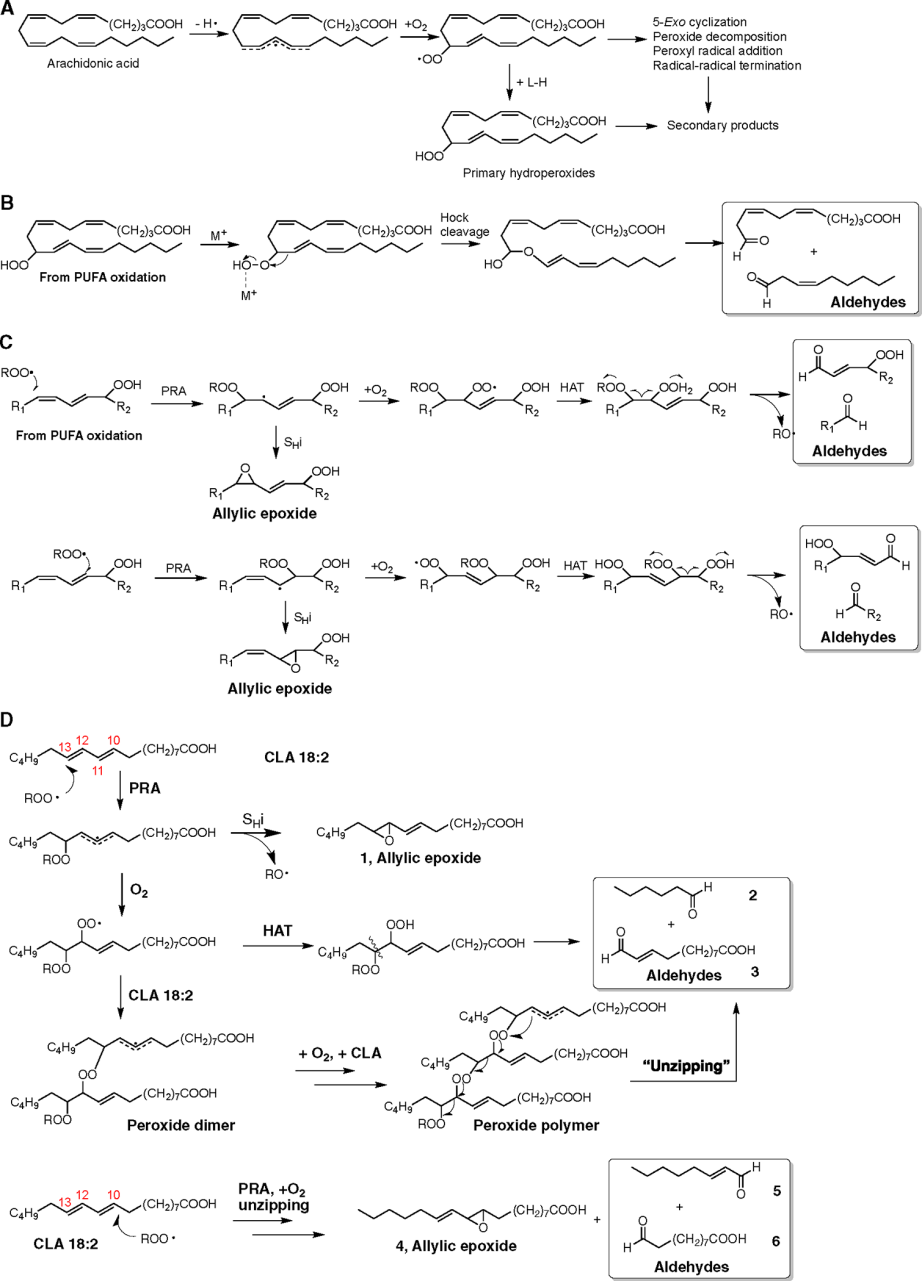
Comparison of products formed from HAT and PRA reaction mechanisms (corrected)

**Figure 3.**
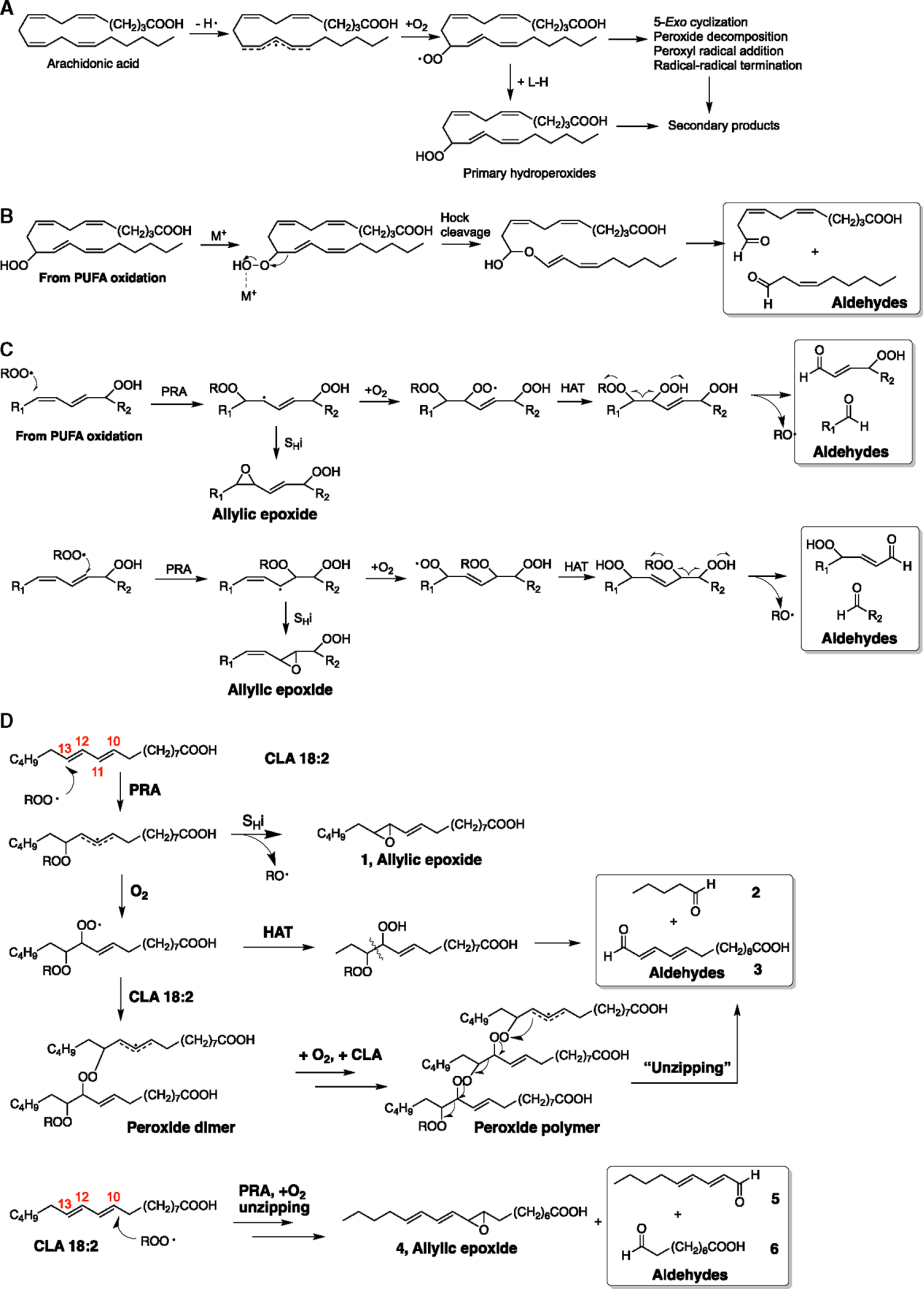
Comparison of products formed from HAT and PRA reaction mechanisms (original)

